# Characteristics and phylogenetic analysis of the complete chloroplast genome of *Lilium concolor* Salisb. (Liliaceae) from Jilin, China

**DOI:** 10.1080/23802359.2021.2006816

**Published:** 2021-12-10

**Authors:** Fengjie Lei, Huanrong Zhang, Yiping Long, Shengkun Deng, Aihua Zhang

**Affiliations:** College of Chinese Medicinal Materials, Jilin Agricultural University, Changchun, PR China

**Keywords:** Complete chloroplast genome, *Lilium concolor*, phylogenetic analysis

## Abstract

*Lilium concolor* Salisb. is a perennial herb with high ornamental and medicinal value in China. The complete chloroplast genome sequence of *L. concolor* was assembled using high-throughput sequencing data. The chloroplast genome of *L. concolor* is 152,625 bp in length and consists of large single-copy (82,056 bp) and small single-copy (17,585 bp) regions, and a pair of inverted repeat (26,492 bp) regions. A total of 131 genes were annotated, these included 85 protein-coding, 38 tRNA, and eight rRNA genes, with an overall GC content of 37.0%. Phylogenetic analysis with 48 chloroplast genomes fully resolved *L. concolor* in a clade with *L. amabile*, *L. callosum*, and *L. pumilum.* This study further confirmed that chloroplast genomes in the genus *Lilium* are highly conserved, which supports the conclusions from previous reports.

*Lilium concolor* Salisb. is a perennial herb classified in Liliaceae (Francis et al. 2004). The plant has upright, star shaped, and dark red small flowers without spots. *Lilium concolor* is an excellent resource for lily breeding in Northeast China, and is favored because of its high ornamental value, strong adaptability, and cold tolerance (Wang et al. 2019). Its flowers and bulbs are rich in protein, amino acids, vitamins, minerals, and other nutrients needed by human body (Wang et al. 2011). Studies show that the main secondary metabolites of *L. concolor* include polysaccharides, saponins, and phenols (Wang et al. 2011). Pharmacological analysis indicates that *L. concolor* possesses many pharmacological activities, mainly including anti-oxidation, anti-bacterial, anti-inflammatory, reducing blood lipid and immune regulation (Zhang et al. 2014; Hou et al. 2016). However, its phylogenetic position remains unclear due to a lack of genomic information. Here, we characterized the complete chloroplast genome sequence of *L. concolor* using high throughput sequencing technology to contribute to the bioinformatics and evolutionary history of *L. concolor* and related species.

The fresh leaves of *L. concolor* were collected from the medicinal botanical garden of Jilin Agricultural University (43°81′N, 125°42′E). Specimens were stored in the Herbarium of Jilin Agricultural University (voucher number LCS210701, Aihua Zhang, blueice20021230@163.com). Total genomic DNA was extracted according to a modified CTAB protocol (Doyle and Doyle 1987). The genome sequencing was performed by Bio&Data Biotechnologies Inc. (Hefei, China) on the BGISEQ-500 platform. The sequences were filtered using fastp (Chen et al. 2018) and then assembled with the SPAdes assembler 3.10.0 (Bankevich et al. 2012). GeSeq (Tillich et al. 2017) and BLASTx (Gish and States 1993) searches were employed for the annotation.

The chloroplast genome of *L. concolor* is a 152,625 bp in length and circular (GenBank accession no. MZ676707). It contains two inverted repeat (IR) regions of 26,492 bp, separated by large single-copy (LSC) and small single-copy (SSC) regions of 82,056 bp and 17,585 bp, respectively. The genome is predicted to have 131 genes, including 85 protein-coding, 38 tRNA, and eight rRNA genes. Five protein-coding, eight tRNA, and four rRNA genes were duplicated in IR regions. Nineteen genes contained two exons and four (*clp*P, *ycf*3, and two *rps*12) contained three exons. The overall GC content of the *L. concolor* cp genome is 37.0% and the corresponding values in LSC, SSC, and IR regions are 34.8%, 30.7%, and 42.5%, respectively. The gene content of the cp genomes of *L. concolor* and three other *Lilium* (*L. amabile*, *L. callosum*, and *L. pumilum*) are nearly identical. *Lilium concolor* has two pseudogenes (*ndh*G and *cem*A), which differs from *L. amabile* and *L. callosum*, which contain only one pseudogene *ndh*G and *L. pumilum* with only one pseudogene, *cem*A (Kim et al. 2017).

Alignment of the *L. concolor* cp genome was performed using complete chloroplast genome sequences along with 48 sequences in the *Lilium* genus (*Cardiocrinum giganteum* and *Fritillaria eduardii* were designated as outgroup taxa) using the autosettings in MAFFT v7.307 (Katoh and Standley 2013). The maximum-likelihood (ML) tree was inferred using the GTR + CAT nucleotide substitution model by FastTree version 2.1.10 (Price 2010). *Lilium concolor* was resolved in a clade with *L. amabile*, *L. callosum*, and *L. pumilum*, with a posterior probability = 1 (Figure 1). The phylogenomic analysis further revealed the high conservation of cp genomes in the genus *Lilium,* which supports the conclusions from a previous analysis (Kim et al. 2017).

**Figure 1.**
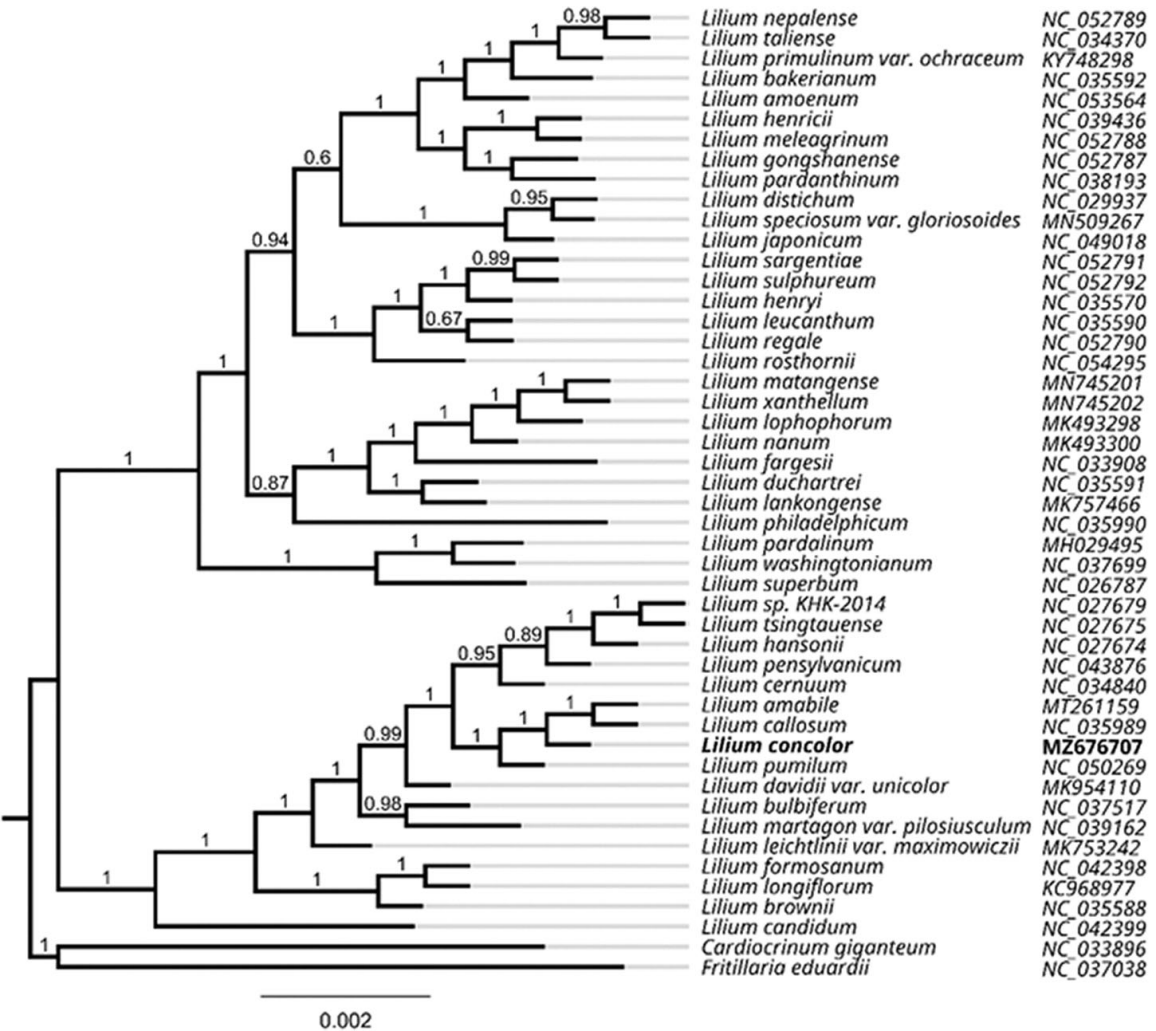
Phylogenetic tree inferred by maximum-likelihood (ML) method based on 48 representative species. *Cardiocrinum giganteum* and *Fritillaria eduardii* were designated as outgroup taxa. The values of posterior probability are shown at the branches. GenBank accession numbers are shown in Figure 1.

## Data Availability

The genome sequence data of *L. concolor* that support the findings of this study are openly available in GenBank of NCBI at https://www.ncbi.nlm.nih.gov/ under the accession no. MZ676707. The associated BioProject, SRA, and Bio-Sample numbers are PRJNA751705, SRR15329922, and SAMN20524810, respectively.
